# Similarities in mindset between adolescents’ friends and cooperation partners

**DOI:** 10.1007/s11218-025-10028-6

**Published:** 2025-02-18

**Authors:** Ilona M. B. Benneker, Nikki C. Lee, Fanny de Swart, Nienke M. van Atteveldt

**Affiliations:** 1 Section of Clinical Developmental Psychology & LEARN! Research Institute, Vrije Universiteit, Amsterdam, The Netherlands; 2 Department of Developmental Psychology, Utrecht University, Utrecht, The Netherlands; 3 Mencia de Mendozalyceum, Breda, The Netherlands

**Keywords:** Mindset, Cooperation partners, Friendship, Adolescents, Networks

## Abstract

Peers, in terms of both friends and cooperation partners, are a very important aspect of the social context of adolescents. They may affect adolescents’ intelligence mindsets and therefore their school motivation and success. Being friends or cooperating with a peer with a similar mindset might either enhance (in case of a growth mindset) or hinder (in case of a fixed mindset) adolescents’ motivation to learn. In this cross-sectional social network study, we first examined whether friendship networks and cooperation partners networks within school classes differ from each other. Second, we investigated whether adolescents’ friends and cooperation partners have similarities in mindsets. We analysed peer nominations and intelligence mindsets within 26 Dutch classes of early and mid-adolescents (N = 558) using the quadratic assignment procedure (QAP). Our data showed that three unique networks could be distinguished: a friendship only network, a combined friends and cooperation partners network and a cooperation only network. Multiple regression quadratic assignment procedures (MRQAP) indicated no evidence for similarity in mindset in all the three networks. However, we did find that adolescents with a growth mindset select more peers to cooperate with than adolescents with a fixed mindset. This latter finding shows that mindset influences social interactions in the context of cooperation between adolescents. It might be valuable to take the social context into consideration in the development of new mindset interventions.

## Introduction

During adolescence, school motivation gradually declines (see e.g. Gnambs & Hanfstingl, 2016; Gottfried et al., 2001; Scherrer & Preckel, 2019). This is worrying as this decline may be detrimental to adolescents’ academic success (Cerasoli et al., 2014; Taylor et al., 2014; Wang et al., 2018) and consequently their career success and health (Archambault et al., 2009). An important determinant of adolescents’ motivation and achievement ( Burnette et al., HYPERLINK "sps:refid::bib11|bib9" ; Sarrasin et al., 2018) is the implicit belief that they hold about the malleability of their intelligence and ability (also called mindset) (Dweck & Molden, 2000). Some researchers challenge the relation between mindset and academic achievement (Sisk et al., 2018), other investigations showed that adolescents who believe that their abilities are malleable (i.e. growth mindset), typically demonstrate higher achievement and more motivation for learning (Benneker et al., 2023; Burnette et al., 2018; Yeager et al., 2019). Given the relation between mindset and adolescents’ academic success, identifying factors that foster mindset is important.

Although mindset is typically approached as an individual trait, the role of the social context in mindset, including the role of peers, has recently gained more attention (de Ruiter & Thomaes, 2023; King, 2020; Lou & Li, 2023; Sheffler & Cheung, 2020). Evidence suggests that adolescents tend to seek out peers who are similar to them with respect to various attributes related to motivation, such as pursuing the same goals and similar levels of self-control (Duriez et al., 2013; Gremmen et al., 2017; Rambaran et al., 2017; Veenstra & Laninga-Wijnen, 2022). Subsequently, peers may reinforce each other in these behaviours. As adolescents spend a large proportion of their time at school, certain classmates, such as the friends or the peers they cooperate with on academic tasks, may be a particularly strong influence on adolescents’ mindset. Being friends or cooperating with a peer with a similar mindset might therefore enhance or hinder adolescents’ motivation to learn (Sheffler & Cheung, 2020).

To date, no studies have investigated similarities in mindset in friendships within classrooms. Moreover, the current literature on similarities between adolescents focusses primarily on friendships and not on cooperation partners, while for school work cooperation partners may be equally important (Roseth et al., 2008). Given the importance of mindset for adolescents’ academic and future career success, identifying how social factors are related to mindset is crucial. Therefore, the overall aim of this study is to gain insight in the role of mindset in friendships and cooperation partners.

### Mindset: an individual or social construct

According to the mindset theory (Dweck & Molden, 2000), mindsets related to intelligence can range from believing that abilities are unchangeable (fixed) and cannot be improved, to believing that they are malleable (growth) and can be improved (Burnette et al., 2013; Dweck & Molden, 2000). There is much empirical research that supports the more adaptive self-regulatory behaviours associated with having a growth mindset (Burnette et al., 2013; Dupeyrat & Mariné, 2005; Dweck & Molden, 2000; Janssen et al., 2022; Renaud-Dubé et al., 2015). For example adolescents with a growth mindset often set learning goals, show mastery-oriented strategies and invest great effort, engagement and persistence in school tasks. Adolescents with a fixed mindset, however, frequently set performance goals, use helpless-oriented strategies and put in less effort and persistence. Other studies show that adolescents with a growth mindset tend to have higher intrinsic motivation (Liu, 2021; Molden & Dweck, 2000; Renaud-Dubé et al., 2015) which remains stable under challenging circumstances (Benneker et al., 2023). These investigations highlight the importance of gaining insight into factors that positively affect the mindsets of adolescents in order to enhance their school motivation and success.

Mindset is primarily approached as an individual characteristic (Burnette et al., 2020), but recent studies suggest that the social context may play an important role as well (de Ruiter & Thomaes, 2023; Kim, 2023; Lou & Li, 2023). A study by Lou and Li (2023) showed that a growth mindset was positively related to performance outcomes on math and reading and this effect was stronger in an environment with growth mindset norms (the extent to which a growth mindset was prevalent within the classroom environment). A similar result was found by Yeager et al. (2019) in their intervention study. When the peer norm was supportive of engaging in academic challenges, the performance outcomes of adolescents in their online growth mindset intervention improved more than in an unsupportive peer context. Furthermore, another study showed that adolescents, in a school environment that was characterized by a growth mindset, experience a greater sense of belonging and achieve higher grades compared to adolescents in a more fixed-oriented school environment (Muenks et al., 2020). These studies suggests that adolescents’ mindset may be affected by characteristics of their social environment. This was shown by King (2020) who found that the mindset of one’s classmate at a specific moment predicted one’s own mindset later on. Moreover, interacting with growth mindset peers was shown to increase task valuing on problem-solving tasks (Sheffler & Cheung, 2020). In their more recent study, Sheffler and Cheung, (2020) showed that being around peers with a growth mindset increased the school performance of adolescents. Thus, peer behaviours may be a meaningful determinant for mindset of adolescents in classrooms. Therefore, it is important to gain more insight in the role of peers in relation to mindset.

### Friendships and cooperation partners

In general, peers are known to be a very important social context for adolescents (Bukowski & Sippola, 2005; Schriber & Guyer, 2016) and they strongly influence each other’s behaviours and motivation (Giletta et al., 2021; Molloy et al., 2011; Wentzel & Muenks, 2016). Given the substantial amount of time spent at school, a significant number of adolescents’ interactions with peers occur within this environment. While at school, adolescents form various types of social relationships. They interact with their friends, but also with classroom peers, for example when cooperating on school tasks. A friendship can be defined as a dyadic close relationship between two peers who provide each other with emotional support and engage in activities and conversations in order to strengthen their emotional connection (Cacioppo & Patrick, 2008; Laursen & Hartup, 2002). In contrast, a cooperation partner is someone chosen by a peer for the exchange of academic work, such as doing a project together or sharing and discussing school material (see for a meta-analysis Johnson & Johnson, 2002).

To fully understand how the peer context is related to mindset, it is crucial to study both adolescents’ friendships and cooperation partners (Hantula & Linkola, 2018; Levy et al., 2004), because adolescents’ friends and cooperation partners may only partly overlap. Ample research shows that adolescents base their friendship choices on similarity in attributes and behaviours, such as gender, religion or sport activities (Cook et al., 2017; Gremmen et al., 2017; Laursen & Hartup, 2002; Osgood et al., 2022). For the selection of cooperation partners, there is some evidence that, within the classroom, adolescents particularly prefer to cooperate with their friends (Kutnick & Kington, 2005; Zander et al., 2019). A previous investigation with late adolescents showed that friendships formed at the beginning of the academic year evolve into cooperation relationships during the academic year (Stadtfeld et al., 2019). These cooperation partners might be based on willingness to contribute to learning during the informally formed friendships (Levy et al., 2004; Stadtfeld et al., 2019; Stallen et al., 2023). However, sometimes adolescents prefer other classmates to cooperate with. Indeed, in their qualitative study, Levy et al. (2004) suggest that adolescents cooperate with others than their friends, implying that adolescents base their choices for friends and cooperation partners on different criteria. For example, a friendship might be based on interest in sports, while a cooperation partner might be chosen on criteria such as reciprocity, taking responsibility and being helpful (Barclay & Van Vugt, 2015; Capraro et al., 2014; Van Ryzin et al., 2020; Wentzel, 2014). This may mean that adolescents in particularly prefer to cooperate with others who are willing and able to confer benefits (Barclay, 2016). These differences in the selection of friends and cooperation partners may affect adolescents’ motivation to learn and are therefore essential to understand.

### Mindset similarity in adolescents’ friends and cooperation partners

Similarity is an important prerequisite in adolescents’ friendship choices. However, not much is known with regard to the role of similarities in mindset. Moreover, it is not clear whether similarity also plays a role in adolescents’ choices for cooperation partners, while cooperation partners might also affect adolescents’ learning, just as their friends. Friendship similarity is often described with Byrne’s similarity attraction theory (for a meta-analysis, see Montoya et al., 2008). According to this theory, adolescents often choose their friends based on similarities, including demographic attributes (e.g., gender), but also on cognitive attributes that are closely related to mindset (e.g., school engagement and self-control) (Gremmen et al., 2017; McPherson et al., 2001; Selfhout et al., 2010; Shin, 2022; Wang et al., 2018). Subsequently, within these friendships, adolescents may reinforce each other’s behaviours through socialisation. Socialisation effects in the school context were found in studies into math anxiety (Kim et al., 2023) and learning languages (Fortuin et al., 2016) showing that friends become more similar over time regarding their levels of math anxiety and their language achievements. Therefore, it might be that similarity in mindset also plays a role in friendships. For the classroom environment, cooperation partners are an equally important peer context.

Although not much is known about similarity in cooperation partners, sociological research into social dilemmas suggests that similarity may also play a role in the choice for cooperation partners. Previous experimental studies showed that adults choose similar others to cooperate with (Melamed et al., 2020; Stallen et al., 2023). For instance, Stallen et al. (2023) indicated that adults choose similarly endowed and productive others to cooperate with, reinforcing disparities within groups. Additionally, an investigation by Melamed et al. (2020) demonstrated that similarity drives the selection of new cooperation partners, thereby increasing clustering in networks. In classrooms adolescents might approach their choices for cooperation partners based on similarity as well, seeking out classmates with similar mindsets who are possibly willing to invest similar levels of effort and persistence. As with friendship, within these selected cooperation partners, socialisation might occur. A recent study, for example, showed that interacting with someone with a growth mindset leads to increases in the extent to which participants valued their own performance on a problem-solving task (Sheffler & Cheung, 2020), showing that participants influence each other. Hence, similarity in cooperation partners could affect learning through these socialisation processes.

It is possible that mindset similarity in both friends and cooperation partners could either hinder or benefit motivation and learning. In case of a fixed mindset, adolescents could reinforce each other’s tendency to invest less effort and persistence in their school work with possible negative consequences for school motivation. At the same time, adolescents with similar growth mindsets may bolster each other’s effort and perseverance in learning which could lead to more motivation for assignments and school tasks. To date, it is however not clear whether similarity in mindset plays a role in adolescents’ friendships and cooperation partners. Therefore, in this study, we investigate the role of similarity in mindset in friendships and cooperation partners.

### Differences between early and mid-adolescents

Similarity in mindset may be important for the selection of friends and cooperation partners in particular during adolescence, and it is therefore important to investigate this specific developmental stage. Adolescence, the period from the age of about ten to the early 20s, is characterized by significant emotional and social changes (Blakemore & Choudhury, 2006; Casey et al., 2008). It is a developmental period in which the amount of time spent with parents decreases, while the amount of time spent with peers increases (Csikszentmihalyi, 2014). This may have an effect on adolescents’ mindset. Even though similarity in mindset may play a role in interactions with friends and cooperation partners for all adolescents, this might be different for early adolescents (10–14 years old) and mid-adolescents (15–19 years old). In adolescence, three developmental phases can be distinguished: early (10–14 years old), mid (15–19 years old) and late (20–24 years old) adolescents (also called emerging adults) (Gentry & Campbell, 2002). Our study focuses on early and mid-adolescents, since there are differences between these two groups regarding their peer interactions. Research shows that early adolescents still show a large amount of flexibility in their peer groups, which is caused both by the transition from primary to secondary school as well as by the cognitive and social changes during these first years of adolescence (Poulin & Chan, 2010; Urdan & Schoenfelder, 2006). Compared to early adolescents, mid-adolescents show an increase in the cohesiveness and quality of friendships (Poulin & Chan, 2010). These differences between early and mid-adolescents may lead to differences in their selection of friends and cooperation partners. In this study, we therefore distinguish between these two developmental phases to gain insight into how mindset plays a role in selection processes of friends and cooperation partners in both phases.

### Current study

In the current cross-sectional, social network study, we aim to investigate the role of mindset in the classroom networks of early and mid-adolescents. Our first research question is “do adolescents select different classroom peers as friends compared to cooperation partners?” We hypothesize that although there will be some overlap between the networks, there will be differences as well (Kutnick & Kington, 2005; Levy et al., 2004). Based on this we tentatively expect that three unique networks may be distinguished: a friendship only network (consisting of peers nominated as friends, but not cooperation partners), a combined friends and cooperation partners network (consisting of friends also nominated as cooperation partners) and a cooperation only network (consisting of peers nominated as cooperation partners but not as friends). Our second research question is “do adolescents choose their peers in these three described networks based on similarities in mindset?” We tentatively hypothesize that adolescents’ friends and cooperation partners may be similar in their mindsets (Gremmen et al., 2017; Kim, 2023) as similarity is a well-known concept for relationships between adolescents to build upon. Therefore peers with similar mindsets are more likely to choose each other as friends and as cooperation partners.

In this second research question, we will also distinguish between early and mid-adolescents, as we expect that choosing friends and cooperation partners based on similarities in mindset is more robust for mid-adolescents compared to early adolescents (Poulin & Chan, 2010; Urdan & Schoenfelder, 2006).

## Methods

### Participants

Participants were Dutch adolescents from two different secondary schools in the Netherlands. We selected adolescents in two distinct developmental phases, early and mid-adolescence. Half of the adolescents was enrolled in year 1 (early adolescents) and the other half in year 4 (mid-adolescents). Adolescents in year 1 are typically 12–13 years old, while adolescents in year 4 are typically 15–16 years old (Centraal bureau voor de Statistiek, 2022).

In total 29 classes participated in the study. Only classes with participation rates of 60% and higher were included in the analysis, which resulted in the exclusion of three classes (*n* = 29). Participation rates lower than 60% may lead to insufficient power and validity of the results of social network analysis (Marks et al., 2013). A total of 26 classes were included in the analysis. A class can be defined as a group of adolescents taking the same lessons at the same time over the course of the school day. Early adolescents (year 1) take all lessons together with their class. For mid-adolescents (year 4) the class comprised the group they took their mandatory subjects with, meaning that they were not with this exact group all lessons. Of these 26 classes, thirteen were year 1 classes (early adolescents) with a total of 278 students (106 boys and 172 girls). The average participation rate of these classes was 83%. The other thirteen classes were year 4 classes (mid-adolescents) with a total of 280 students (110 boys and 170 girls). The average participation rate per year 4 class was 84%. The average classroom size for both year 1 and year 4 was 26 students (see Table 1 for the sample descriptives and see appendix A for the descriptives per class).

**Table 1:** Descriptive statistics of the participants

Variables	Year 1 (early adolescents)	Year 4 (mid-adolescents)
Participants (% of total)	278 (49.82%)	280 (50.18%)
Number of classes	13	13
Average classroom size (range)	26 (range: 16–32)	26 (range: 22–31)
Average participation rate	82.67%	82.62%
Average age participants*	12–13 years	15–16 years
Girls (%): Boys (%)	106 (38.13%): 172 (61.87%)	110 (39.29%): 170 (60.71%)
Level of education** (% of total)		
Vmbo-t / havo***	54 (19.42%)	NA
Havo	33 (11.87%)	2158 (56.43%)
Havo / vwo	49 (17.63%)	NA
Vwo	142 (51.08%)	122 (43.57%)
Average mindset *(SD*)	4.81 (.71)	4.64 (.73)****

^*^ According to Statistics Netherlands (CBS) (2022)

^**^ The Dutch schooling system in secondary school is divided into levels of education based on academic performance. Vmbo-t (Lower General Secondary Education) prepares adolescents for further vocational training. There are two levels which prepare adolescents for higher education – Havo (Higher General Secondary Education) and Vwo (Pre-University Education)

^***^ Year 1 often has classes with combined levels of education, such as Vmbo-t / havo. This allows adolescents to get used to secondary education and choose their level of education in the second or third year. Year 4 never has classes with combined levels of education

^****^ *p* < *.05 (t-test)*

### Procedure

Prior to the study, the researchers contacted secondary schools interested in participating in scientific research. Schools that agreed to participate were asked to forward an email with information about the research project to first and fourth year students and their parents / guardians. Adolescents and their parents who wanted to participate gave written informed consent. All procedures were approved by the Ethical Review Board of the Vrije Universiteit Amsterdam, Faculty of Behavioural and Human Movement Sciences. In May and June 2019 adolescents completed the questionnaires via their smartphone during a lesson under the supervision of their mentor and the researcher. Adolescents were seated separately and were unable to see each other’s responses. They did not receive any incentive for their participation.

### Measures

#### Friendships and cooperation partners

Friendships and cooperation partners were assessed using the peer nomination procedure (Cillessen & Marks, 2017; Newcomb & Bukowski, 1983). Participants were shown a list of their classmates, and asked to select peers from the list. Adolescents who did not give consent also were not passively included in the social network questionnaire (this meant that adolescents could not select non-participating class mates as friends or as cooperation partners).The peer nominations included questions about friends (i.e. ‘Who are your friends in class?’) and a question about cooperation partners (i.e. ‘Who would you like to cooperate with on a school task?’). Participants could nominate an unlimited number of peers for these two categories, but self-nominations were not allowed.

We used the nominations for the item ‘Who are your friends in class?’ to construct the social network (friendship network), whereas the nominations for the item ‘With whom would you like to cooperate on a school task?’ were used to construct the academic network (cooperation network).

#### Mindset

Mindset was measured by utilizing the Implicit theories of Intelligence Scale (Dweck & Molden, 2000) adapted by De Castella and Byrne (2015) and translated to Dutch by van Aalderen-Smeets et al. (2019). The scale consists of four fixed mindset items and four growth mindset items. Answers were scored on a Likert-scale from 1 to 6 in which 1 meant ‘strongly disagree’ and 6 ‘strongly agree’. The four fixed mindset items were reverse scored and the mean of the eight items was calculated. A higher score reflected a stronger growth mindset, while a lower score reflected a stronger fixed mindset. An example of a growth mindset statement is ‘Regardless of my current intelligence level, I think I have the capacity to change it quite a bit’ and an example of a fixed mindset statement is ‘To be honest, I don’t think I can really change how intelligent I am’. Validity of the Dutch version of this scale was checked in previous work and the (combined fixed and growth) mindset scale was found to be of good validity (van Aalderen-Smeets et al., 2019). Cronbach’s alpha for internal consistency showed satisfactory reliability (α = 0.78) in the current sample.

### Analysis

To answer the research questions, social network analyses were conducted in R 4.0.3 (Team, R. C. & others, 2018). In social network analysis, observations are interdependent instead of independent and therefore statistical methods such as linear regression are not applicable (Ernst & Albers, 2017). Observations in networks can affect each other through for example reciprocity and transitivity, and are often part of the same social system (in our investigation: schools). Taking into account that observations are interdependent, we used a quadratic assignment procedure correlation (QAP correlation) for the first research question on the differences between the friendship and cooperation networks. For the second research question on mindset similarities, we used a multiple regression quadratic assignment procedure (MRQAP) (Dekker et al., 2007; Krackhardt, 1988). We conducted these analyses with the netglm package (Elmer, 2021).

#### Friendships and cooperation partners

To investigate the differences between friends’ and cooperation partner networks, we conducted QAP procedures (Team, R. C. & others, 2018). In QAP correlation, whole networks are reshaped into two long columns and Pearson’s correlation coefficient is then calculated. To calculate the significance of this observed correlation, the rows and columns of one of the networks were permuted and correlated with the other network, repeating this process thousands of times to calculate how often the random correlation is greater than or equal to the observed correlation. The random permutation of the networks generated a reference null distribution that controls for network structure and therefore provides a solution for the violation of independence assumption. A low proportion (< 0.05) indicated a strong relationship between the two networks (Borgatti et al., 2013). We calculated densities of these different networks and t-tests were conducted to compare these densities.

#### Mindset similarity in adolescents’ friends and cooperation partners

Next, to investigate the role of mindset similarity in the different networks, three multiple regression quadratic assignment procedure (MRQAP) were used to investigate associations between characteristics of dyad (e.g., mindset similarity) and a binary tie variable (e.g., being friends or cooperation partners). This was done separately for the three different networks (ties in the networks are the dependent variables): friends only, combined friends and cooperation partners and cooperation only. Independent variables were presented in the form of matrices representing predictors. These predictors were mindset sender, mindset receiver and mindset similarity. The term sender referred to the adolescent nominating other adolescents and the mindset sender effect is the associations between the mindset of the adolescent and the tendency to nominate others, irrespective of their self-reported mindset. The term receiver referred to the adolescent who is nominated by other adolescents. This captured the associations between the mindset of an adolescent and the tendency to be nominated, irrespective of the nominators self-reported mindset. Mindset similarity referred to the absolute score difference in mindset between two adolescents in a class. Control variables were gender similarity and study year (early adolescents (year 1) or mid-adolescents (year 4).

In line with MRQAP procedures, four steps were followed. In step 1 a classic regression (logistic or linear) was run with ties in the networks as the dependent variables and predictors of interest (e.g., similarity) as independent variables. The level of analysis was the dyadic relationship between two adolescents in the network and not the individual. These regression coefficients were then stored, since only the estimated standard errors are influenced by the assumption of independence. In step 2 the network with the dependent variable was permuted, which means that both row and columns were rearranged simultaneously. This is considered the most conservative method to obtain statistical inference. This way a large set of networks (in our analysis 2000) were created with on average no association between the dependent network and the independent variables. In step 3 the first step is repeated with the permuted networks. Lastly, in step 4, regression coefficients from step 3 (the null distribution) were compared with the regression coefficients from step 1. It is then possible to compute the 97.5% and 2.5% percentiles to obtain a measure similar to the 95% confidence interval to provide information about the range in which the true value lies. Additionally, we also calculated a p-value by assessing how many coefficients from the null distribution are larger or equally large than the observed coefficient. We applied a multilevel extension of the MRQAP in order to take the multilevel data structure (class level) in to account. In the analysis, all levels were analysed simultaneously, but different from the general MRQAP is that the permutations (see step 3) were carried out within each class. We included gender similarity as a covariate, as gender similarity is an important prerequisite for friendship selection and socialisation (McMillan, 2022; Smetana et al., 2006). Furthermore, to assess whether mindset similarity affected selection of friends and cooperation partners differently for early and mid-adolescents, an interaction term between mindset similarity and study year was included in the models.

## Results

### Friendships and cooperation partners

**Fig. 1:**
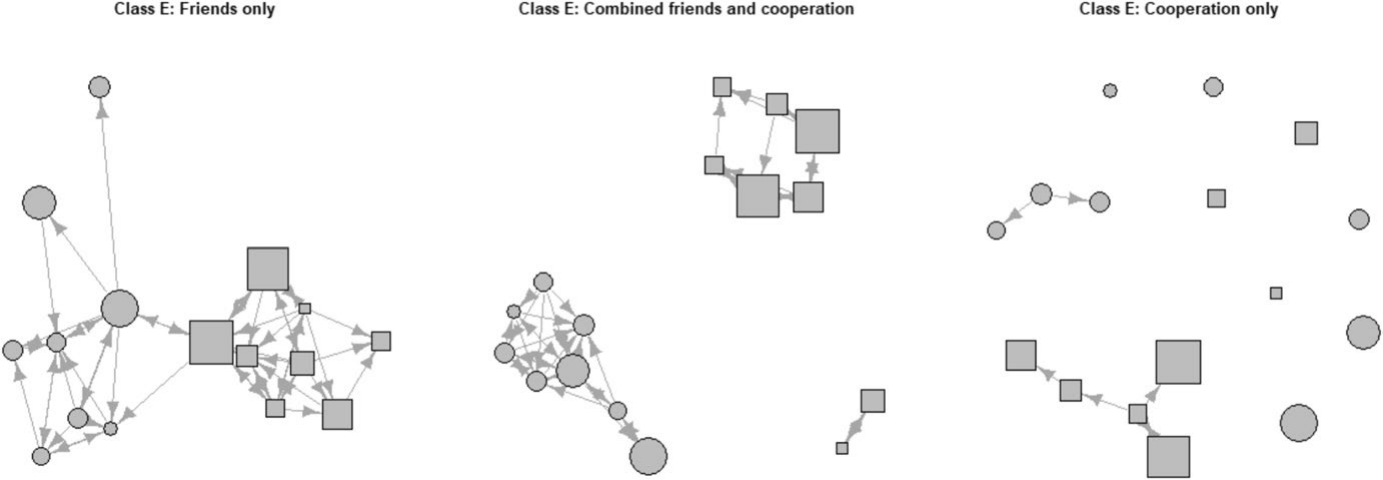
Example of three network plots of the same class (class E), *N* = 16 (year 1 early adolescents). Circles = female adolescents, squares = male adolescents. Size of node represents score on mindset. Larger nodes have a more growth mindset, smaller nodes have a more fixed mindset. Arrows represent the direction of ties

The first research question was whether there were differences between the adolescents’ friendship networks and cooperation partners networks. The results showed a significant correlation between the friendship and cooperation partners networks in all 26 classes with an average correlation coefficient of 0.52 (ranging from 0.38 to 0.63) (see Appendix B for the QAP correlations per class), which can be considered as a moderate correlation (Mukaka, 2012). Based on these distinct differences in correlations, we identified three networks with unique ties (dependent variables) in all three networks. The first network consisted of adolescents who were friends with each other, but not cooperation partners (friendship only network). The second network consisted of friends who also like to cooperate with each other (combined friends and cooperation partners network). The third network consisted of peers who were preferred cooperation partners, but not friends (cooperation only network). To assess the proportion of ties in relation to the possible relationships in these three networks (the total amount of actual relationships divided by the total amount of possible relationships in a class), the density of the networks was calculated. The friendship only networks had an average density of 0.19 (*SD* = 0.06) with an average number of 89 ties per class, the combined friends and cooperation partners networks had an average density of 0.17 (*SD* = 0.04) with an average number of ties of 80 per class, and the cooperation only networks had an average density of 0.03 (*SD* < 0.01) with an average number of ties of 12 per class (see Appendix C for densities and ties per class). To illustrate this, Fig. 1 shows three network plots of one randomly selected class. The figure shows the higher density for the combined friendship and cooperation networks and the friends only networks compared to the cooperation only networks. This means that there are more relationships between peers in both the friends only and the combined friends and cooperation partners networks as compared to the cooperation only networks. Network figures for all other classes can be found in Appendix D.

In order to get a better understanding of the differences between the early and mid-adolescents, we first did an initial analysis on the difference between mindset in early and mid-adolescents. A two sample t-test showed that mid-adolescents had a significant lower score on mindset (*M* = 4.64, *SD* = 0.73), hence reported a more fixed mindset, compared to early adolescents (*M* = 4.81, *SD* = 0.71), *t*(555.83) = 2.90, *p* = 0.004. Next, we analysed the differences between the early and mid-adolescents networks. Three paired-samples t-tests were performed to compare the densities of the friends only networks, the combined friends and cooperation partners networks and the cooperation only networks in early adolescents (year 1) and mid-adolescents (year 4). The friendship only networks showed a significant difference in density between early adolescents (*M* = 0.22, *SD* = 0.06) and mid-adolescents (*M* = 0.17, *SD* = 0.06); *t*(12) = − 2.94, *p* = 0.012 with higher densities for early adolescents, which means that early adolescents have more friends only connections than mid-adolescents do. No significant differences were found between early adolescents (*M* = 0.17, *SD* = 0.04) and mid-adolescents (*M* = 0.18, *SD* = 0.04) for the combined friends and cooperation partners networks; *t*(12) = − 0.31, *p* = 0.762. Additionally, the cooperation only networks showed a significant difference in density between early (*M* = 0.01, *SD* = 0.01) and mid (*M* = 0.04, *SD* = 0.02) adolescents as well; *t*(12) = − 2.86, *p* = 0.014 with lower densities for early adolescents. This means that early adolescents have fewer cooperation only partners compared to mid-adolescents.

### Mindset similarity in adolescents’ friends and cooperation partners

In Table 2 the results of the analyses regarding the friendship only networks are depicted. No significant effect was found for similarity in mindset (*b* = − 0.05, *p* = 0.342), indicating that adolescents do not base their friendships on similarity in mindset. We found a significant effect for gender similarity (*b* = − 0.61, *p* < 0.001), indicating that adolescents often nominated friends of the same gender. We also found no significant interaction between study year and mindset similarity (*b* = − 0.02, *p* = 0.465). Early and mid-adolescents did not differ in selecting their friends based on similarities in mindsets. No significant effects were found for mindset sender (*b* = − 0.05, *p* = 0.163), mindset receiver (*b* = − 0.02, *p* = 0.177) and study year (*b* = − 0.32, *p* = 0.465).

**Table 2:** Results of multiple regression quadratic assignment procedure model for the friendship only networks

Variables	*b*	*p*	E (Est)**	*Percentiles*	
				2,5th	97,5th
Intercept	− 0.32	0.012*	− 1.08	− 1.82	− 0.37
Mindset sender	− 0.05	0.163	0.01	− 0.10	0.12
Mindset receiver	− 0.02	0.177	0.01	− 0.07	0.08
Mindset similarity	− 0.05	0.342	0.02	− 0.28	0.33
Gender similarity	− 0.61	0.000*	0.01	− 0.10	0.12
Study year	− 0.32	0.307	− 0.27	− 0.43	− 0.12
Mindset similarity x Study year	− 0.02	0.465	− 0.03	− 0.23	0.17

^*^p < .05

^**^E(Est) = Expected value of the estimate under the null distribution (i.e. the mean value of regression coefficients in the permuted scenarios)

Bold values indicate statistical significance at *p* < .05

In Table 3 the results of the analyses regarding the combined friends and cooperation partners networks are depicted. Comparable to the friendship only networks, we found no significant effect for similarity based on mindset (*b* = − 0.28, *p* = 0.055). Adolescents did not base their selection of friends with whom they prefer to cooperate on having the same mindset. The results indicated a significant effect of gender similarity on the presence of a tie between adolescents (*b* = − 1.55, *p* < 0.001), which means that adolescents often named friends with whom they prefer to cooperate who also had the same gender as themselves. We also found no significant effect for the interaction term mindset similarity x study year (*b* = 0.17, *p* = 0.065). Early and mid-adolescents did not differ in choosing their friends with whom they prefer to cooperate based on having a similar mindset. The results showed a significant effect for study year (*b* = − 0.14, *p* = 0.032) when other variables were constrained to zero. Mid-adolescents compared to early adolescents selected fewer friends with whom they prefer to cooperate with. Additionally, no significant effects were found for mindset sender (*b* = 0.05, *p* = 0.195) or mindset receiver (*b* = 0.05, *p* = 0.142).

**Table 3:** Results of multiple regression quadratic assignment procedure model for the overlapping friends and cooperation partner networks

Variables	*b*	*p*	E (Est)**	*Percentiles*	
				2,5th	97,5th
Intercept	− 1.29	0.116	− 1.68	− 2.36	− 1.04
Mindset sender	0.05	0.195	0.01	− 0.08	0.10
Mindset receiver	0.05	0.142	0.01	− 0.06	0.08
Mindset similarity	− 0.28	0.055	− 0.02	− 0.33	0.28
Gender similarity	− 1.55	0.000*	0.01	− 0.11	0.13
Study year	− 0.14	0.032*	0.01	− 0.14	0.16
Mindset similarity x Study year	0.17	0.065	0.02	− 0.17	0.21

^*^p < .05

^**^E(Est) = Expected value of the estimate under the null distribution (i.e. the mean value of regression coefficients in the permuted scenarios)

Bold values indicate statistical significance at *p* < .05

Table 4 presents the results of the cooperation only networks. We found no significant effect for similarities in mindset (*b* = 0.20, *p* = 0.476) and gender (*b* = − 0.15, *p* = 0.180). Adolescents do not select cooperation partners based on having the same mindset. Furthermore, boys do not necessarily select boys, and girls do not necessarily select girls to cooperate with. Their selection for cooperation seems to be more heterogeneous. We also found no significant effect for the interaction term mindset x study year (*b* = − 0.05, *p* = 0.438), indicating that there is no difference in naming cooperation partners based on having the same mindset between early and mid-adolescents. We also found no significant effect on study year (*b* = 0.88, *p* = 0.490) or on mindset receiver (*b* = − 0.12, *p* = 0.273). The findings indicate a significant positive sender effect for mindset (*b* = 0.18, *p* = 0.047). Adolescents with a growth mindset named more peers with whom they prefer to cooperate than adolescents with a fixed mindset.

**Table 4:** Results of multiple regression quadratic assignment procedure model for the cooperation only networks

Variables	*b*	*p*	E (Est)**	*Percentiles*	
				2,5th	97,5th
Intercept	− 5.34	0.125	− 4.32	− 6.04	− 2.65
Mindset sender	0.18	0.047*	− 0.07	− 0.34	0.22
Mindset receiver	− 0.12	0.273	− 0.08	− 0.22	0.06
Mindset similarity	0.20	0.476	0.16	− 0.68	0.98
Gender similarity	− 0.15	0.180	− 0.03	− 0.30	0.24
Study year	0.88	0.490	0.88	0.52	1.27
Mindset similarity x Study year	− 0.05	0.438	− 0.08	− 0.55	0.38

^*^p < .05

^**^E(Est) = Expected value of the estimate under the null distribution (i.e. the mean value of regression coefficients in the permuted scenarios)

Bold values indicate statistical significance at *p* < .05

**Fig. 2:**
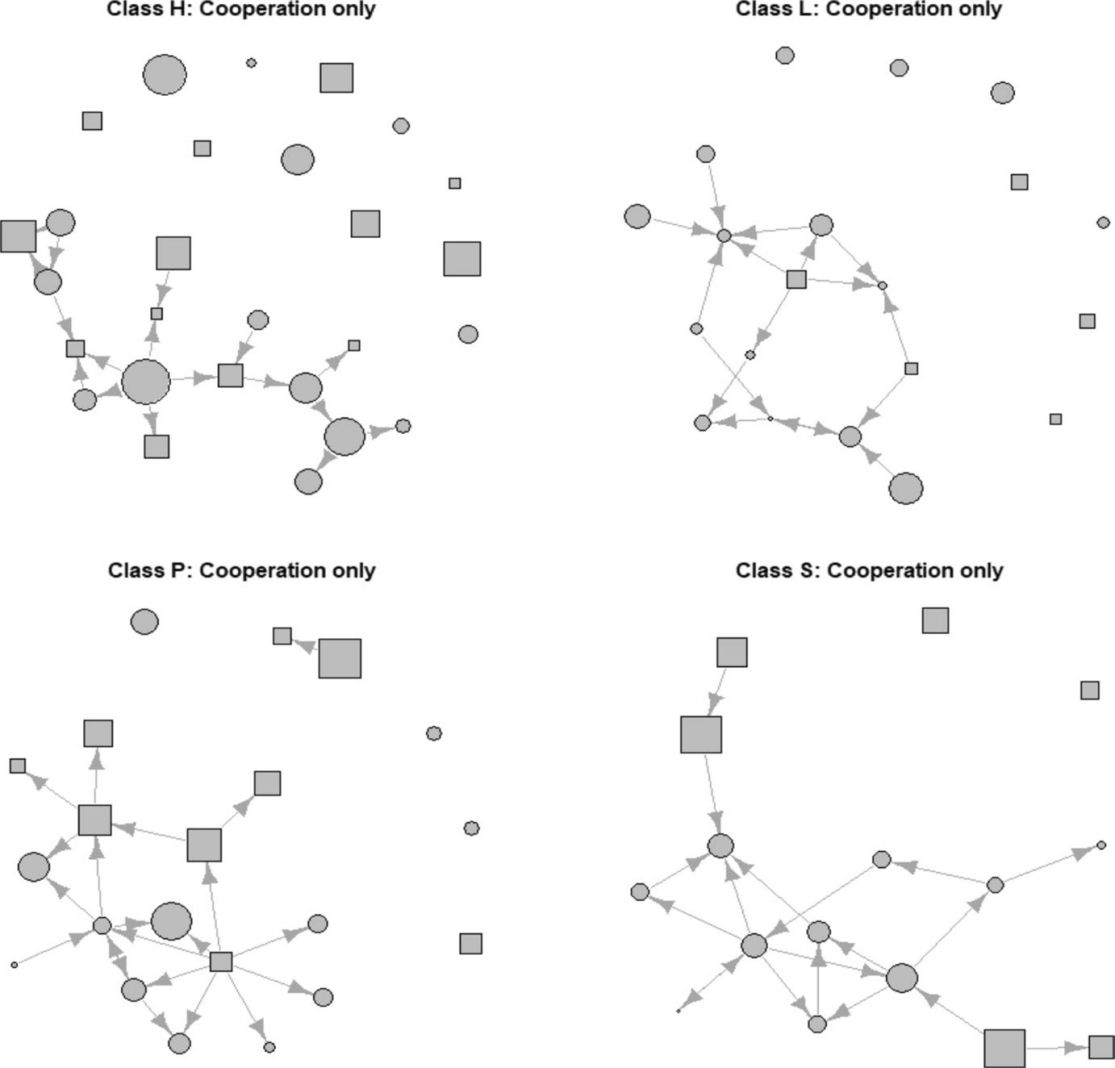
Plots of various cooperation only networks. Class H (*N* = 27) and L (*N* = 20) are year 1 classes. Class P (*N* = 21) and S (*N* = 16) are year 4 classes. Circles = female. squares = male. Size of node represents score on mindset. Larger nodes have a more growth mindset. Smaller nodes have a more fixed mindset

Figure 2 shows this result visually. The figure shows that adolescents with a more growth mindset (larger circles (female) and squares (male) nominate more peers with whom they prefer to cooperate compared to adolescents with a more fixed mindset (smaller circles (female) and squares (male)).

## Discussion

In this study, we aimed to investigate the role of mindset in adolescents’ friendships and cooperation partners. First, we identified whether adolescents chose the same or different peers as their friends and as their cooperation partners. Second, we examined whether these friends and cooperation partners were similar to each other in mindsets. In line with our first hypothesis, our findings indicated that three distinct networks could be identified: a friendship only network, a combined friends and cooperation partners network and a cooperation only network. This suggests that while adolescents often prefer to cooperate with their friends on schoolwork, some choose their cooperation partners based on other criteria as well. Contrary to our second hypothesis, within these three networks, we did not find evidence for similarities in mindsets between adolescents in either early or mid-adolescents. We did find that, within the cooperation only network, adolescents with a growth mindset named more peers to cooperate with than adolescents with a fixed mindset did. We discuss each of the findings and their implication in more detail below.

**Table 5:** Descriptive statistics of participants of year 1 (early adolescents) and year 4 (mid-adolescents) per class: total participants. participation rate. class size and gender are shown

	Total participants (participation rate)	Class size	Boys (%): Girls (%)
Year 1 (early adolescents)			
Class A	15 (93.75%)	16	8 (53.33%): 7 (46.67%)
Class B	23 (82.14%)	28	9 (39.13%): 14 (60.87%)
Class C	26 (96.30%)	27	10 (38.46%): 16 (61.54%)
Class D	17 (85.00%)	20	7 (41.18%): 10 (58.82%)
Class E	16 (84.21%)	19	8 (50.00%): 8 (50.00%)
Class F	27 (96.43%)	28	13 (48.15%): 14 (51.85%)
Class G	24 (88.89%)	27	12 (50.00%): 12 (50.00%)
Class H	27 (84.38%)	32	13 (48.15%): 14 (51.85%)
Class I	18 (69.23%)	26	5 (27.78%): 13 (72.22%)
Class J	18 (62.07%)	29	4 (22.22%): 14 (77.78%)
Class K	18 (64.29%)	28	6 (33.3%): 12 (66.67%)
Class L	20 (71.43%)	28	5 (25.00%): 15 (75.00%)
Class M	29 (96.67%)	30	6 (20.69%): 23 (79.31%)
Year 4 (mid-adolescents)			
Class N	18 (78.26%)	23	3 (16.67%): 15 (83.33%)
Class O	21 (87.50%)	24	14 (66.67%): 7 (33.33%)
Class P	21 (70.00%)	30	9 (42.86%: 12 (57.14%)
Class Q	21 (87.50%)	24	10 (47.62%): 11 (52.38%)
Class R	24 (88.89%)	27	4 (16.67%): 20 (83.33%)
Class S	16 (64.00%)	25	6 (37.50%): 10 (62.50%)
Class T	21 (91.30%)	23	14 (66.67%): 7 (33.33%)
Class U	19 (86.36%)	22	10 (52.63%): 9 (47.37%)
Class V	25 (86.21%)	29	5 (20.00%): 20 (80.00%)
Class W	22 (75.86%)	29	4 (18.18%): 18 (81.82%)
Class X	28 (100.00%)	28	11 (39.29%): 17 (60.71%)
Class Y	23 (74.19%)	31	8 (34.78%): 15 (65.22%)
Class Z	21 (84.00%)	25	12 (57.14%): 9 (42.86%)

**Table 6:** Quadratic Assignment Procedure correlation (QAP correlation) to compare the friendship network with the cooperation network

	Coefficient	t	df	p	95% CI
Year 1 (early adolescents)					
Class A	.600	11.074	223	< .001	.504–.067
Class B	.522	14.071	527	< .001	.458–.582
Class C	.526	16.037	674	< .001	.469–.578
Class D	.479	9.237	287	< .001	.385–.563
Class E	.506	9.342	254	< .001	.408–.592
Class F	.589	19.657	727	< .001	.540–.635
Class G	.463	12.509	574	< .001	.396–.525
Class H	.564	18.416	727	< .001	.512–612
Class I	.514	10.766	322	< .001	.430–.590
Class J	.424	8.397	322	< .001	.330–.509
Class K	.490	10.099	322	< .001	.403–.569
Class L	.488	11.163	398	< .001	.410–.560
Class M	.589	21.088	839	< .001	.543–.631
Year 4 (mid-adolescents)					
Class N	.474	9.662	322	< .001	.385–.554
Class O	.538	13.387	439	< .001	.469–.602
Class P	.597	15.582	439	< .001	.533–.654
Class Q	.491	11.536	418	< .001	.415–.561
Class R	.639	19.903	574	< .001	.588–.685
Class S	.573	11.143	254	< .001	.484–.650
Class T	.399	9.120	439	< .001	.318–.475
Class U	.514	11.341	359	< .001	.433–.586
Class V	.455	12.767	623	< .001	.391–.515
Class W	.480	12.020	482	< .001	.409–.546
Class X	.517	16.884	782	< .001	.464–.566
Class Y	.628	18.509	527	< .001	.573–.677
Class Z	.581	14.960	439	< .001	.516–.640

**Table 7:** Densities and ties of the thirteen year 1 networks and the thirteen year 4 networks

	Friendship and cooperation network density (ties)	Friendship only network density (ties)	Cooperation only network density (ties)
Year 1 (early adolescents)			
Class A	0.209 (47)	0.218 (49)	0.000 (0)
Class B	0.136 (72)	0.217 (115)	0.004 (2)
Class C	0.161 (109)	0.240 (162)	0.003 (2)
Class D	0.266 (77)	0.304 (88)	0.014 (4)
Class E	0.160 (41)	0.191 (49)	0.027 (7)
Class F	0.167 (122)	0.185 (135)	0.005 (4)
Class G	0.168 (97)	0.264 (152)	0.016 (9)
Class H	0.173 (126)	0.167 (122)	0.023 (17)
Class I	0.179 (58)	0.253 (82)	0.006 (2)
Class J	0.173 (56)	0.327 (113)	0.009 (3)
Class K	0.123 (40)	0.173 (56)	0.025 (8)
Class L	0.175 (70)	0.185 (74)	0.043 (17)
Class M	0.147 (124)	0.126 (106)	0.027 (23)
Year 4 (mid-adolescents)			
Class N	0.148 (48)	0.219 (71)	0.022 (7)
Class O	0.177 (78)	0.211 (93)	0.014 (6)
Class P	0.168 (74)	0.104 (46)	0.048 (21)
Class Q	0.188 (83)	0.168 (79)	0.052 (24)
Class R	0.196 (113)	0.130 (75)	0.024 (14)
Class S	0.262 (67)	0.125 (32)	0.074 (19)
Class T	0.259 (114)	0.313 (138)	0.036 (16)
Class U	0.172 (62)	0.172 (62)	0.039 (14)
Class V	0.141 (88)	0.142 (89)	0.064 (40)
Class W	0.144 (70)	0.171 (83)	0.039 (19)
Class X	0.102 (80)	0.136 (107)	0.022 (17)
Class Y	0.164 (87)	0.147 (78)	0.009 (5)
Class Z	0.175 (77)	0.127 (56)	0.041 (18)

### Friendships and cooperation partners

We distinguished three unique networks, namely a friendship only network, a combined friends and cooperation partners network and a cooperation only network. Previous experimental and observational research showed that adolescents tend to be better at cooperating with friends than with nonfriends (Rubin, 2011). However, this does not provide much insight into the extent to which adolescents also prefer to cooperate with friends and thus to what degree these networks overlap. In line with our expectations, we found that while adolescents often tend to choose their friends as cooperation partners, there are some exceptions. Some peers are only chosen as either a friend or as a cooperation partner. This suggests that adolescents might utilize different selection criteria for their friends and their cooperation partners. Criteria that could play a role are, for example, the extent to which the potential friend or cooperation partner shows certain prosocial behaviours, such as helping each other, taking responsibility and being benevolent. These behaviours have been known to be related to both friendship (Beffel & Neal, 2023; Cillessen et al., 2005) and cooperation behaviours (Capraro et al., 2014; Van Ryzin et al., 2020; Wentzel, 2014). It is possible that some prosocial behaviours may be more important for the selection of cooperation partners than for the selection of friends. For example, taking responsibility for a task might be more important for a cooperation partner than for a friend, while in a friendship supporting each other might be more appreciated. However, more research is needed to find out to what extent these prosocial behaviours may be important criteria for adolescents to base their selection of friends or cooperation partners on.

Another important difference between the social networks was that adolescents’ altogether nominated more classmates as friends (higher density) than cooperation partners. An explanation for this may be that friendships are often based on similarities in various attributes, such as gender, religion or sport activities (Gremmen et al., 2017; Shin, 2022). For example, adolescents may have some friends with the same gender and some other friends who share the same religion. Additionally, they may also have a few friends with whom they play sports. Friendships therefore may be based on this large variety of attributes. Contrarily, cooperation partners are only chosen to do school assignments with and there may be fewer attributes to choose these cooperation partners on. This may explain in particular the numerous relations in the friendship only networks compared to the cooperation only networks. Conform expectations, there was also considerable overlap between classmates that were considered friends and cooperation partners (density almost equal to friends only network). This may be explained by the tendency of friends to help and (emotionally) support each other, which often leads to reciprocal behaviour and may thereby also create a foundation for cooperation (Majolo et al., 2006; Rubin et al., 2011). This is in line with Stadtfeld et al. (2019) who demonstrated that newly formed friendships between older adolescents at the beginning of the academic year evolve into cooperation relationships during the academic year. Another explanation may be that peers prefer to cooperate with their friends and that they select a specific subsample of their friends to cooperate with. It is possible that the specific subsample of cooperation partners consist of friends who have specific characteristics relevant within an academic context, such as high levels of school engagement or conscientiousness, that make cooperation feasible. The fact that cooperation partners are preferably friends is confirmed by the few ties (low density) of adolescents with classmates in the cooperation only networks.

Importantly, our findings suggested differences in friendships of early and mid-adolescents. First, early adolescents scored significantly higher on mindset, hence had a more growth mindset, than mid-adolescents. A possible explanation for this might be that adolescents, as they go through education, may realize that to continue the same level of education, performance and achievements become more important than the process of learning. Adolescents may adjust their mindset towards this more performance-oriented situation (de Ruiter & Thomaes, 2023), resulting in a more fixed mindset when they reach mid-adolescence and a subsequent decrease in motivation. Previous studies showed that adolescents who have a growth mindset, tend to have higher motivation (Liu, 2021; Molden & Dweck, 2000; Renaud-Dubé et al., 2015). A growth mindset may therefore be an important protective factor for the decline in adolescents’ motivation and consequently their academic success. Our findings underline the importance of different mindset interventions depending on the phase of adolescence. Interventions for early adolescents could for example focus more on maintaining a growth mindset, while interventions for mid-adolescents could focus more on fostering a growth mindset.

Second, early adolescents had more friends than mid-adolescents. A possible explanation might be that mid-adolescents have fewer, but more intense friendships, due to the fact that mid-adolescents seek more cohesiveness in their friendship compared to early adolescents who still show a large amount of flexibility in their peer groups (Poulin & Chan, 2010; Urdan & Schoenfelder, 2006). A second explanation for this is that the mid-adolescents in our sample might have more friends outside their own class than early adolescents have, due to the fact that in the Dutch educational system mid-adolescents only follow a selection of classes together with the same classmates compared to early adolescents who follow all classes together. It is therefore likely that mid-adolescents also have some friends in other classes. In addition, we found that in the cooperation only networks mid-adolescents reported more cooperation partners than early adolescents. It is possible that early adolescents may often select their friends as cooperation partners (Levy et al., 2004) to be able to practice cooperation within the safe environment of their friendships. Mid-adolescents, on the other hand, may already have more experience with social relationships (Rubin et al., 2011) and therefore may be less focussed on their friends for cooperation. Other criteria may become more important when selecting cooperation partners. This finding seems to be consistent with a previous investigation by Swenson and Strough (2008), who showed that mid-adolescents performed equally well on a scientific reasoning task when they cooperated with a friend or with a non-friend. This indicates that cooperation with friends in this age group is not necessarily beneficial. It is possible that mid-adolescents consider other characteristics, such as school engagement, when choosing their cooperation partners.

### Mindset similarity in adolescents’ friends and cooperation partners

We investigated to what extent adolescents choose their friends and cooperation partners based on similarity in mindset. Contrary to our expectations, we could not find any evidence for adolescents having friends or cooperation partners with similar mindsets. This suggests that selection and socialisation processes that play a role in related areas such as school engagement and self-control (Gremmen et al., 2017; McPherson et al., 2001; Selfhout et al., 2010; Shin, 2022; Wang et al., 2018) may not be applicable to mindset. On one hand, this may be positive, since this limits the risk for negative socialisation processes. For example, if two fixed mindset adolescents do not choose each other as friends or as cooperation partners, they will also not reinforce each other’s mindset and thereby do not bolster each other to demonstrate less effort and persistence which may lower their school motivation. On the other hand, this result means that adolescents might not positively influence each other with these positive behaviours. For example, if two growth mindset adolescents do not choose each other as friends or as cooperation partners, they also may not shape each other’s mindset. This may then not affect their effort and persistence in school tasks and therefore may not lead to more school motivation.

A possible explanation for the fact that mindset does not seem to play a role in adolescents’ friendship and cooperation networks may be that mindset is not be a visible characteristic. Adolescents’ own mindsets may shape their goal orientation and self-regulatory behaviours (Burnette et al., 2013; Dweck & Molden, 2000; Janssen et al., 2022), but their beliefs may only be noticed by peers if it leads to visible actions (Haimovitz & Dweck, 2016). In addition to this, adolescents with similar mindsets may be a heterogeneous group with substantial differences in their motivations (Yu & McLellan, 2020) and other aspects of these motivations may be more visible for adolescents and therefore a stronger selection criterium. For example, in their investigation, Yu and McLellan (2020) distinguished four motivational profiles. Two of these profiles supported the classic mindset theory: a group of adolescents had a growth mindset and mastery goal orientation, another group had a fixed mindset and performance goal orientation. However, they also identified two other profiles. Some growth mindset adolescents displayed concurrent performance goals, while some fixed mindset adolescents did not hold performance goals. Their results indicated that the relation between mindset and other motivational constructs such as goal orientation may be more nuanced than previously indicated. Goal orientation has previously been linked to strategies such as a mastery-oriented strategy or a helpless-oriented strategy. Adolescents with a mastery-oriented strategy show persistence and tenacity when facing challenges, while adolescents with helpless-oriented strategies often show diverted attention and procrastination (Burnette et al., 2013). These learning strategies may be noticeable for other adolescents and as a consequence goal orientation may be more visible than mindset. Consequently, selection and socialisation processes might occur on other processes than mindset. This may have influenced our non-significant results on mindset similarities between adolescents in the three different networks.

Although our findings did not reveal mindset similarity effects, we did observe several other findings. First, we found that adolescents prefer to be friends with those of the same gender as themselves. This does not only account for their friends, but also for friends who they selected as cooperation partners as well. This is in line with other literature that shows that adolescents often prefer to be friends with someone with the same gender (McPherson et al., 2001). Interestingly, we did not find this result in the cooperation only network, indicating that selecting peers with the same gender may be particularly important for friendships. Cooperation partners may not be based on gender and this mixed gender cooperation could even have a positive effect as indicated by a recent investigation, in which the best outcome in a collaborative task was achieved between two individuals with opposite genders (Cigarini et al., 2020). Another possibility is that for the selection of cooperation partners, seating arrangement may play a role. Previous studies showed that seating arrangements in class affects engagement (Bolden et al., 2019; Fernandes et al., 2011) and class participation (Fernandes et al., 2011; Lotfy, 2012). Adolescents may select their cooperation partners based on previous experiences they had with their classmates when they were seated next to them or when they worked together in groups assigned by the teacher. These selection criteria may be more important than gender.

Another interesting finding was that adolescents with a growth mindset had more cooperation partners than adolescents with a fixed mindset. This is in line with other studies that show that adolescents with a growth mindset often focus on learning and endorse a mastery goal orientation (Burnette et al., 2013; Dweck & Molden, 2000) and those adolescents are often more willing to cooperate with others regardless of the beliefs of the other (Levy et al., 2004; Marijn Poortvliet et al., 2007). This may mean that adolescents with a growth mindset find cooperation with peers important as they may find it valuable to acquire new knowledge and skills and may therefore search for a wider range of cooperation partners. Simultaneously, adolescents with a more fixed mindset may limit their cooperation partners as they only want to cooperate with those they consider to be able to perform well on a task or whom they consider to be ‘smart’. Additionally, adolescents who are able to cooperate well with others have more positive relationships with peers and have higher achievements (Johnson et al., 2010; Roseth et al., 2008). Therefore, our findings provide some evidence that the social component should be included when developing and implementing interventions that promote a growth mindset.

### Strengths and limitations

Our study had several strengths. This study is the first to investigate mindset within friends and cooperation partners networks, as previous research mainly focussed on mindset as an individual trait. Second, not only did we consider the friendship network of adolescents, but we investigated their cooperation network as well as adolescents spend a large proportion of their time in school where they often work together on school tasks. Third, we had a large and representative sample, as we were able to include 26 almost complete classes in our analyses and we could distinguish between early and mid-adolescents.

However, a number of limitations should also be noted with regards to the current study. First, we used a cross-sectional design. Therefore, we were unable to disentangle peer selection from socialisation (influence) processes. Future longitudinal studies will be helpful to disentangle these two processes and provide us with more insight on how the mindset of an adolescent may influence the mindset of a peer, friend or cooperation partner, over the course of a school year. An initial study by King (2020) already demonstrated that the mindset of one’s classmate at the beginning of the school year predicted one’s own mindset seven months later.

Second, our study may not have considered other variables that could potentially help to understand the role of mindset within social networks. A future investigation could for example focus on different motivational profiles, in which the same mindset might be related to different goal orientations and related strategies, in order to see mindset as a part of a motivational belief system, as indicated by Yu and McLellan (2020).

Third, we collected network nominations within classrooms. It was therefore not possible for adolescents to indicate relations that they had with other adolescents within the same school or within the same year group. This might be especially important for mid-adolescents, who do not take all lessons with the same peers and therefore may have their friends and cooperation partners in other classes as well. It is possible that this limited selection possibility may have resulted in fewer friendship and cooperation ties within class and this may have influenced our results on similarity in mindsets between adolescents.

Fourth, previous investigations revealed that socioeconomic status may play a role in the relation between mindset and achievement (Claro et al., 2016; Destin et al., 2019). For example, Claro et al. (2016) demonstrated that holding a growth mindset may buffer against the negative effects of poverty on achievement. The schools that participated in our study were located in a very affluent region in a medium-sized city in the southern part of the Netherlands (CBS, 2022). Therefore, our findings may be less generalizable to adolescents with different socioeconomic backgrounds. Therefore, without further research, we cannot generalise our current findings. More work with a more diverse sample is needed to fully understand the role of mindset similarity in early and mid-adolescents with other socioeconomic backgrounds.

## Conclusion

In summary, this study examined the role of mindset in the social context of early and mid-adolescents. Based on the differences in adolescents’ friends and cooperation partner selection, we identified three distinct networks: a friendship only network, a combined friends and cooperation network and a cooperation only network. We did not find any effect of peer nominations based on similarities in mindset for either early or mid-adolescents within all three types of networks. Interestingly, we found that adolescents with a growth mindset choose more cooperation partners compared to adolescents with a fixed mindset. This finding is important, because adolescents who are able to cooperate with different other adolescents show more positive peer relations and higher academic achievements. Our findings provide some first evidence to include the social context of adolescents when new growth mindset interventions are developed and implemented.

## Appendix A

See Table 5.

## Appendix B

See Table 6.

## Appendix C

See Table 7.
